# Dietary Supplementation With Fine-Grinding Wheat Bran Improves Lipid Metabolism and Inflammatory Response *via* Modulating the Gut Microbiota Structure in Pregnant Sow

**DOI:** 10.3389/fmicb.2022.835950

**Published:** 2022-03-24

**Authors:** Zijie Wang, Yifan Chen, Wenhui Wang, Caiyun Huang, Yongfei Hu, Lee Johnston, Fenglai Wang

**Affiliations:** ^1^State Key Lab of Animal Nutrition, College of Animal Science & Technology, China Agricultural University, Beijing, China; ^2^College of Animal Science and Technology, Hebei Agricultural University, Baoding, China; ^3^College of Animal Science, Fujian Agriculture and Forestry University, Fuzhou, China; ^4^Swine Nutrition and Production, West Central Research and Outreach Center, University of Minnesota, Morris, MN, United States

**Keywords:** fine-grinding wheat bran, gut microbiota, lipid metabolism, inflammation, pregnant sow

## Abstract

This study investigated the effects of fine-grinding wheat bran on pregnant sow body condition, lipid metabolism, inflammatory response, and gut microbiota. In this study, wheat bran was crushed into three particle sizes. A total of 60 Landrace × Yorkshire second parity sows were allotted to two groups: CWB (a diet containing coarse wheat bran with particle size of 605 μm) and FWB (a diet containing fine wheat bran with particle size of 438 μm). Fine-grinding wheat bran had higher soluble dietary fiber concentration, swelling capacity, water-holding capacity, and fermentability than coarse wheat bran. Pregnant sows fed FWB throughout pregnancy had lower body weight and fat deposition than sows fed CWB. And the piglet body weight at birth of the FWB group was remarkably increased. Serum concentrations of lipids (triglycerides, total cholesterol, and free fatty acid), interleukin 6, leptin, and resistin were decreased on day 90 of pregnancy by fine wheat bran supplementation. Feeding FWB significantly decreased abundance of *Firmicutes* and dramatically increased the abundance of *Bacteroidetes* at phylum level. At genus level, the abundance of *Terrisporobacter* was decreased in FWB feeding sows, but the abundance of *Parabacteroides* was increased. Fecal total short-chain fatty acids, propionate, and butyrate contents were markedly increased in the FWB group. The results suggested that the physicochemical properties of finely ground wheat bran had been improved. Dietary supplementation with fine wheat bran changed the gut microbiota structure and enhanced the short-chain fatty acids level, which improved the maternal body condition, metabolic and inflammatory status, and reproductive performance in sows.

## Introduction

In recent years, maternal obesity during pregnancy has become an epidemic problem. Excessive pregnancy weight gain or obesity caused by diet might have a significant impact on maternal health and outcome of the pregnancy and might have long-lasting influences on the descendants (Cedergren, 2004; Challier et al., 2008). Maternal obesity is related to modifications in the placental transcriptome, which increases placental lipid content, oxidative stress, and inflammation (Kim et al., 2014; Saben et al., 2014). Gut microbiota plays considerable roles in host physiology (Tuohy et al., 2012). The microbial composition of obese patients has been changed, mainly increased in *Firmicutes* and decreased in *Bacteroidetes* (Ley et al., 2006; Dahiya et al., 2017). In addition, gut microbiota is correlated with weight gain, inflammatory profiles, and lipids parameters of pregnant women (Santacruz et al., 2010; Houttu et al., 2017).

Dietary fiber (DF) includes polysaccharides and lignin which are not digested by host intestinal endogenous enzymes (Gouseti et al., 2019). Supplementation of DF is an effective measure to maintain body health, including reduced risk of obesity (Kim et al., 2016) and associated complications such as cardiovascular disease (Erkkila and Lichtenstein, 2006) and type II diabetes (InterAct Consortium, 2015). DF is the energy substance of microbiota residing in the cecum and colon, which affects the composition, diversity, and richness of gut microbiota (Makki et al., 2018). Metabolism of DF by gut microbes yields a large number of short-chain fatty acids (SCFAs), which impact the gut microbial ecology, physiology, and health of the organism (Ríos-Covián et al., 2016). Differing physicochemical properties of DF elicit significantly different physiological effects in the host (Brownlee, 2011). Fine grinding of cereals decreases particle size and increases surface area compared with coarse grinding. Increased surface area improves solubility, swelling capacity (SC), and water-holding capacity (WHC) (Li et al., 2016). Wheat bran can reduce the expression of proinflammatory factors (Jiminez et al., 2016) and ameliorate lipid profiles in C57BL/6J mice fed with a high-fat diet (Han et al., 2015). But wheat bran is a cereal-derived DF with a high concentration of insoluble dietary fiber (IDF) that has limited physiological effects in the host.

There are few studies aimed to improve the physiological effects of wheat bran through decreasing particle size and to evaluate how fine-grinding wheat bran might influence gut microbiota to improve sow health and reproductive performance. We hypothesize that physicochemical properties of fine-grinding wheat bran have been improved, and adding it to the diet during pregnancy may reduce adiposity, improve maternal lipid metabolism, and decrease inflammation by altering the composition, diversity, and richness of gut microbes. Consequently, the present study was aimed at (1) exploring the physicochemical properties of wheat bran ground to different particle sizes (2) and investigating the effects of dietary wheat bran with varying particle sizes on the composition and metabolites of fecal microorganism as well as pregnant sow lipid and inflammation parameters.

## Materials and Methods

### Ethics Approval

All experimental protocols in the present research were approved by the Animal Care and Use Committee of China Agricultural University (AW420202-2-1) and performed under the National Research Council’s Guide for the Care and Use of Laboratory Animals.

### Wheat Bran Samples

Wheat bran was purchased from Hebei Flour Mill, a shredding machine equipped with 1.0, 1.5, or 2.0-mm screen (Jiangyin Hongda Powder Equipment Limited Company, Wuxi, China), was used to grind wheat bran into three different particle sizes.

### Determination of Particle Size

After grinding, the 14-sieve method of the ANSI/ASAE S319.4-2008 standard (Stark and Chewning, 2011) was used to determine the geometric mean particle size of samples.

### Dietary Fiber Content Testing

Total dietary fiber (TDF), soluble dietary fiber (SDF), and IDF concentrations of wheat bran samples were measured using the method described by Prosky et al. (1988). β-Glucan and arabinoxylan were assayed using gas-liquid chromatography as described by Knudsen (1997).

### Determination of Swelling Capacity and Water-Holding Capacity

One unground and three ground wheat bran samples were analyzed for SC and WHC as described by Canibe and Knudsen (2002) and Serena and Bach Knudsen (2009).

### Animals and Diets

Experiments were implemented at the Fengning Swine Research Unit of China Agricultural University. One week before breeding, Landrace × Yorkshire second parity sows (*n* = 60) were assigned randomly within weight (164.12 ± 3.15 and 165.04 ± 3.07 kg, respectively) and backfat thickness (15.15 ± 0.26 and 15.15 ± 0.26 mm, respectively) to one of two experimental diets. Sows were inseminated artificially with Duroc boar semen on the day of estrus and 24 h after estrus. From days 16 to 21 after insemination, sows were considered to be in estrus when they having a standing reflex in front of the boar. Pregnancy was confirmed using B-model ultrasonic device on the 30th day after insemination. Sows found not pregnant were eliminated from the experiment. Beginning 1 week after breeding, sows dwelled individually in gestation stalls (2.1 m × 0.7 m) and were transferred to farrowing crates (2.1 m × 1.8 m) 1 week before delivery. The average ambient temperature in the gestation house was maintained at 22–26°C. All piglets were born naturally without induction.

During gestation, all sows were fed twice daily at 8:00 and 14:00. From mating to day 30 of gestation, all sows received 2.2 kg/day, and from days 31 to 90 of gestation, sows were fed 2.5 kg/day. From days 91 to 110, sows were offered 3.0 kg daily of their assigned diet, and sows had free access to water throughout the experiment. Feed allowance was reduced gradually from 3.0 to 2.5 kg/day in the 4 days immediately before parturition. The experimental diets included early and middle (from mating to day 90 of gestation) and late pregnancy (from day 91 of pregnancy until farrowing) feed (Supplementary Table 1). The CWB (a diet containing coarse wheat bran with particle size of 605 μm) and FWB (a diet containing fine wheat bran with particle size of 438 μm) have the same level of wheat bran (20%). All experimental diets met or exceeded nutrient requirements of pregnant sows (National Research Council, 2012).

Sows were weighed on days 90 and 107 of gestation and on the day after farrowing. Backfat thickness of each sow was measured at the level of the last rib on the left side 6.5 cm from the midline of the back using ultrasound scanner (Mylab touch, Italy) on these same days. Piglets were weighed at birth.

### Blood, Tissue, and Fecal Sample Collection

The blood of sows was collected on days 90 and 107 of pregnancy after fasting for one night and then feeding for 4 h. Blood was collected from sows by ear vein, and umbilical cord blood was collected from piglets after delivery. All blood samples (5 ml) were collected in tubes without anticoagulant and allowed to clot for 1 h at room temperature. Clotted blood was centrifuged for 15 min at 3,000 × *g*. Serum was collected and stored at −20°C for analysis of lipids, proinflammatory factors, and adipocytokines. Expelled placenta was collected immediately after sow delivery. Five to six samples of placental membrane and connective tissues were collected from the same middle position of each placenta and instantly stored at −20°C for subsequent analysis of lipids, proinflammatory factors, and adipocytokines. Fresh feces were collected on day 90 of gestation, frozen in liquid nitrogen, and stored at −80°C for analysis of microbiota composition and SCFAs.

### Nutrient Contents of Diets

Diet samples were ground through a 1-mm screen and then analyzed for dry matter (DM) (Association of Official Analytical Chemists [AOAC], 2007; method 930.15), crude protein (CP) (Association of Official Analytical Chemists [AOAC], 2007; method 976.05), and ash (Association of Official Analytical Chemists [AOAC], 2007; method 942.15). Diet samples were analyzed for neutral detergent fiber (NDF), TDF, SDF, and IDF by using the method described by Prosky et al. (1988).

### Biochemical Analysis

Total cholesterol (TC), triglyceride (TG), and free fatty acid (FFA) concentrations of maternal serum, placenta, and cord blood were determined by using a commercially available kit (Lebotry Technology Development Limited Company, Beijing, China).

### Detection of Adipocytokines Level

The levels of leptin (LEP), resistin, and adiponectin (ADPN) in serum were determined using ELISA kit (Lebotry Technology Development Limited Company, Beijing, China). Concentrations of interleukin 6 (IL-6), interleukin 8 (IL-8), and tumor necrosis factor-α (TNF-α) in maternal serum, placenta, and cord blood were assayed using an ELISA kit (Lebotry Technology Development Limited Company, Beijing, China).

### Bacterial Microbiota Analysis

Microbiota composition was assayed using tag-encoded 16s rRNA gene MiSeq-based (Illumina) high-throughput sequencing. Bacterial DNA was isolated from sow’s fecal samples using the DNA Kit. The V3-V4 hypervariable regions of the bacterial 16S rRNA gene were amplified using primers F338 (5′-ACTCCTACGGGAGGCAGCAG-3′) and R806 (5′-GGACTACHVGGGTWTCTAAT-3′). The PCR conditions were pre-degenerate 3 min at 95°C, degenerate 27 cycles at 95°C, anneal for 30 s at 55°C, elongation at 72°C for 30 s, and finally extension for 10 min at 72°C. Amplicons were extracted from the 2% agarose gel, and AxyPrep DNA Gel Extraction Kit was used for purification. Amplicons were quantified using QuantiFluor-ST (Promega, Madison, United States). Purified amplicons were combined at equimolar concentrations and paired-end sequenced on Illumina MiSeq platform (2 × 300).

### *In vitro* Fermentation

Fermentability of wheat bran ground to different particle sizes was detected according to the methods of Pastell et al. (2009). Four samples of different particle sizes of wheat bran were fermented in fecal batch cultures. Batch culture fermentation vessels (300-ml capacity: one container for each treatment) were sterilized and filled with 135 ml of sterile basic medium [g/l: 2 g peptone, 2 g yeast extract, 0.1 g NaCl, 0.04 g K_2_HPO_4_, 0.04 g KH_2_PO_4_, 0.001 g MgSO_4_⋅7H_2_O, 0.01 g CaCl_2_⋅6H_2_O, 2 g NaHCO_3_, 2 ml Tween 80, 0.05 g hemin (dissolved in a few drops of 1 M NaOH), 10 μl vitamin K, 0.05 g L-cysteine HCl, 0.5 g bile salts, and 4 ml of 0.02% resazurin solution]. The sterile medium was ventilated overnight with O_2_-free N_2_ (15 ml/min) to establish anaerobic conditions. To simulate the pH of the anterior segment of the sow’s large intestine, pH was maintained in the range of 6.7–6.9 by adding 0.5 mol/L NaOH or 0.5 mol/L HCl automatically through the pH meter controller. The temperature of the culture was maintained at 37°C.

Fecal samples were gathered from six healthy pregnant sows on day 90 of gestation. Feces were pooled, diluted (1:6) with 0.9% NaCl solution, homogenized, filtered through a double thickness of cheesecloth, and added to each fermentation vessel (5 ml) under anaerobic conditions.

Three grams wheat bran samples were put into a fermentation vessel that contained (1) wheat bran, not ground (Control); (2) wheat bran 1 ground through a 2.0-mm sieve (Large); (3) wheat bran 2 ground through a 1.5-mm sieve (Medium); and (4) wheat bran 3 ground through a 1.0-mm sieve (Small). Samples of the fermentation broth (5 ml) were collected after 24 h of fermentation and stored at −80°C until they were analyzed.

### Short-Chain Fatty Acids Analysis

Short-chain fatty acids concentrations of fermentation broth and feces were assayed after extraction according to the method described by Pastell et al. (2009). In brief, fermentation broth samples (400 μl) were injected into the apparatus, and then methanol and acetonitrile (600 μl, respectively) were added to the apparatus and mixed. The mixture was centrifuged at 12,000 × *g* for 15 min, then the supernatant was diluted 10 times with distilled water, filtered, and added to detection bottles. The mobile phase was a sulfuric acid aqueous solution (2.5 mM); flow rate was 0.6 ml/min, and the peak integration was performed with Agilent ChemStation software (Agilent Technologies, Chongqing, China) using a single-point internal standard method. Peak identity and quantification were determined using a mixture of standards of acetic, butyric, iso-butyric, propionic, lactic, formic, valeric, and isovaleric acids. For feces, 0.5 g of feces were suspended in 8.0 ml of distilled water and centrifuged at 5,000 × *g* for 10 min at 4°C. Then the supernatant was diluted 50 times with distilled water. The other steps were the same as in the determination of SCFAs content in fermentation broth.

### Statistical Analysis

Data were analyzed using the general linear model (GLM) procedure of SAS (version 9.2; SAS Inst. Inc., Cary, NC, United States), with sow as the experimental unit. Means were separated using the Tukey test. Data are presented as means ± SEM. Figures were created using GraphPad Prism (version 5; GraphPad Software Inc., San Diego, CA, United States). Community composition of microbiota and diversity with standardized operational taxonomic unit (OTU) readings was analyzed using R software (version 3.3.1; R Software Inc., Auckland, New Zealand). The relative abundances of microbiota composition at the phylum and genus levels were analyzed by Fisher’s exact test. Correlations between SCFAs, lipids, proinflammatory factors, adipocytokines, and bacterial taxa were analyzed using Spearman’s correlation analysis. *p* < 0.05 was considered statistically significant, and tendencies were declared at 0.05 ≤ *p* < 0.10.

## Results

### Particle Size, Swelling Capacity, and Water-Holding Capacity

Particle size decreased with decreasing pore size of the screen used for grinding (Table 1). In comparison with Control, the particle size of the Large, Medium, and Small groups was smaller (*p* < 0.01). Also, differences among the Large, Medium, and Small groups were very significant (*p* < 0.01). With reduction of particle size, WHC and SC increased in the Medium and Small groups. Specifically, WHC in the Medium and Small groups was significantly greater than that in the Control and Large groups (*p* < 0.01). SC in the Small and Medium groups were significantly greater than that of the Control and Large groups (*p* < 0.01).

**TABLE 1:** Particle size, swelling capacity, and water-holding capacity of wheat bran with different particle sizes.

Item	Control	Large	Medium	Small	SEM	*p*-value
Particle size (μm)	605^a^	528^b^	461^c^	438^d^	4.39	<0.001
Water-holding capacity (g/g)	5.17^b^	5.19^b^	6.04^a^	6.13^a^	0.03	<0.001
Swelling capacity (ml/g)	2.07^b^	2.14^b^	2.59^a^	2.60^a^	0.01	<0.001

*Mean values in the same row with different letters are different (p < 0.05). SEM, standard error of the mean (n = 4); Control, no ground wheat bran; Large, wheat bran ground through a 2.0-mm sieve; Medium, wheat bran ground through a 1.5-mm sieve; Small, wheat bran ground through a 1.0-mm sieve.*

### Content and Composition of Dietary Fiber

**FIGURE 1:**
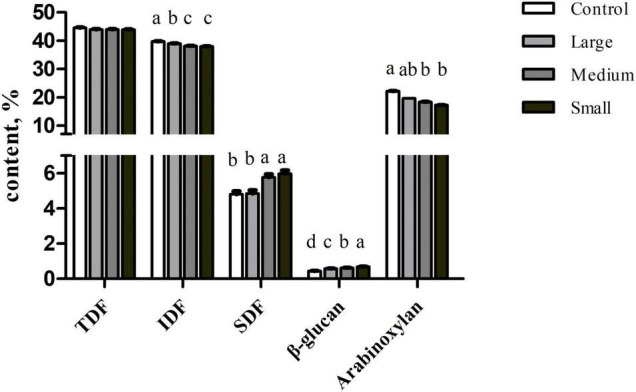
Contents of TDF, SDF, IDF, β-glucan, and arabinoxylan of wheat bran with different particle sizes, %. Data are shown as mean ± SEM (*n* = 4); ^a–d^represents significant difference among different treatments (*p* < 0.05); Control, no ground wheat bran; Large, wheat bran ground through a 2.0-mm sieve; Medium, wheat bran ground through a 1.5-mm sieve; Small, wheat bran ground through a 1.0-mm sieve; TDF, total dietary fiber; SDF, soluble dietary fiber; IDF, insoluble dietary fiber.

The TDF content was similar among samples (*p* > 0.05; Figure 1). But the SDF content was significantly increased in the Small group than that in the Control and Large groups (*p* < 0.05). In contrast, IDF level decreased in the Small group compared with that in the Control and Large groups (*p* < 0.05). To further explore the compositional changes in soluble and insoluble components, we measured β-glucan and arabinoxylan content. We observed that β-glucan concentration increased (*p* < 0.01) with the reduction of particle size. But compared with Control, arabinoxylan content decreased in the Small group (*p* < 0.05).

### Body Weight Gain and Backfat Thickness of Sows During Pregnancy

On days 90 and 107 of pregnancy, FWB feeding led to a lower body weight and backfat thickness compared to CWB (*p* < 0.05) (Table 2). Similarly, FWB decreased weight gain and the gain of backfat thickness throughout pregnancy compared to CWB (*p* < 0.05).

**TABLE 2:** Effect of feeding sows a gestation diet containing different particle sizes of wheat bran on sow body condition during gestation.

Item	CWB	FWB	*p*-value
No. of sows	30	30	–
Parity	2	2	–
**Sow BW, kg:**			
Breeding	161.8 ± 2.72	159.7 ± 2.61	0.57
Gestation, day 90	230.3 ± 2.84	221.0 ± 1.98	0.01
Gestation, day 107	259.9 ± 3.13	239.8 ± 3.47	<0.01
Farrowing, within 24 h	210.3 ± 2.35	201.4 ± 2.44	0.80
BW gain during pregnancy	48.8 ± 0.22	42.1 ± 1.82	0.04
**Sow backfat thickness^a^, mm:**			
Breeding	15.2 ± 0.3	15.6 ± 0.3	0.74
Gestation, day 90	18.3 ± 0.3	17.3 ± 0.3	<0.01
Gestation, day 107	22.5 ± 0.2	19.9 ± 0.2	<0.01
Farrowing, within 24 h	22.7 ± 0.3	20.1 ± 0.2	<0.01
Backfat change throughout pregnancy	7.4 ± 0.1	4.5 ± 0.2	<0.01

*CWB, a diet containing coarse wheat bran, with particle size of 605 μm; FWB, a diet containing fine wheat bran, with particle size of 438 μm; BW, body weight. Data are shown as mean ± SEM.*

*^a^The last rib is 6.5 cm from midline of the back.*

### Reproductive Performance of Sows

There was no difference in total litter size at birth, number of stillborn piglets, and farrowing duration between the CWB and FWB groups (*p* > 0.05) (Table 3). Compared with the CWB group, the born alive litter size and birth weight of piglets was significantly increased in the FWB group (*p* < 0.05). And the birth weight of piglets was higher in the FWB group than that in the CWB group (*p* < 0.05).

**TABLE 3:** Effect of feeding sows a gestation diet containing different particle sizes of wheat bran on sow’s reproductive performance.

Item	CWB	FWB	*p*-value
No. of sows	30	30	–
Parity	2	2	–
**Litter size at birth**			
Total birth	15.11 ± 0.41	14.73 ± 0.22	0.35
Born alive	12.87 ± 0.31	13.80 ± 0.38	0.03
No. of stillborn piglets	0.15 ± 0.09	0.11 ± 0.06	0.73
Farrowing duration, h	5.15 ± 0.38	4.73 ± 0.22	0.35
Piglet BW at birth, kg	1.34 ± 0.02	1.41 ± 0.03	0.04

*CWB, a diet containing coarse wheat bran, with particle size of 605 μm; FWB, a diet containing fine wheat bran, with particle size of 438 μm; BW, body weight. Data are shown as mean ± SEM.*

### Lipid Levels in Maternal Blood, Placentae, and Cord Blood

**FIGURE 2:**
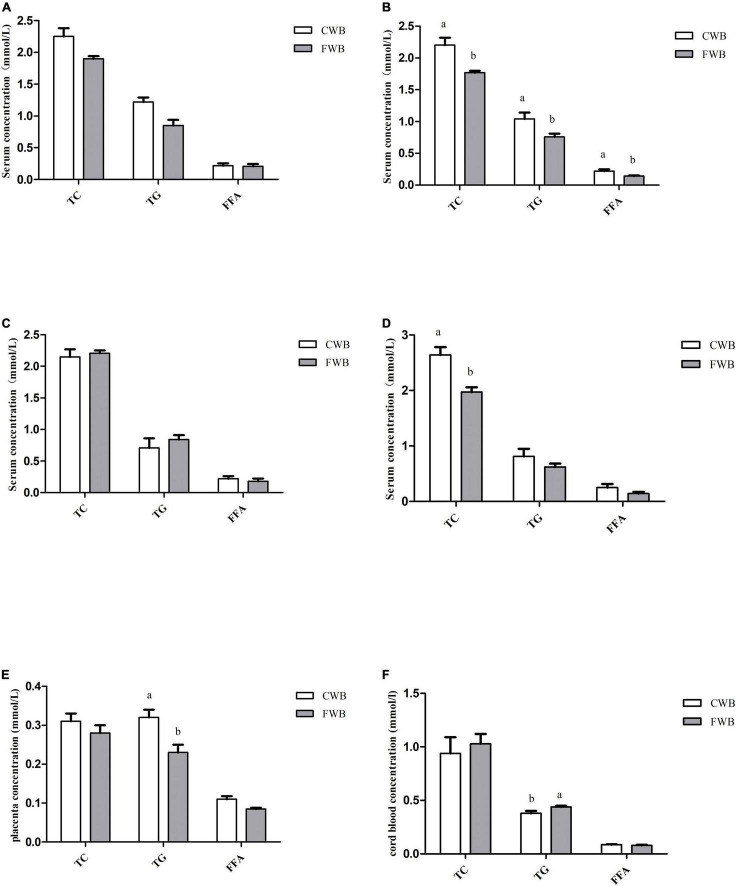
Effects of different particle sizes of wheat bran on levels of TC, TG, and FFA in maternal serum, placentae, and cord blood. **(A)** Fasting serum on day 90 of pregnancy. **(B)** Serum at 4 h after feeding on day 90 of pregnancy. **(C)** Fasting serum on day 107 of pregnancy. **(D)** Serum at 4 h after feeding on day 107 of pregnancy. **(E)** Placenta. **(F)** Cord blood. CWB, a diet containing coarse wheat bran, with particle size of 605 μm; FWB, a diet containing fine wheat bran, with particle size of 438 μm; TC, total cholesterol; TG, triglycerides; FFA, free fatty acid. Values are presented as mean ± SEM (*n* = 8). ^a,b^Means without common letters differ at *p* < 0.05.

During gestation days 90 and 107, fasting serum levels of TG, TC, and FFA were not affected by different particle sizes of wheat bran (Figures 2A,C). However, after a feeding diet of 4 h on day 90 of pregnancy, sows fed FWB diet had lower (*p* < 0.05) level of TG, TC, and FFA than sows fed CWB diet (Figure 2B). And likewise, the concentration of TC in the serum of the FWB group was significantly lower than that of the CWB group on day 107 of pregnancy (*p* < 0.05) (Figure 2D). In addition, FWB-fed sows had lower level of TG (*p* < 0.05) in the placenta than that from CWB-fed sows (Figure 2E). Nevertheless, compared with CWB-fed sows, the TG content in cord blood was increased (*p* < 0.05) in FWB-fed sows (Figure 2F).

### Levels of Adipocytokines in Maternal Serum

During gestation, fasting serum levels of LEP and ADPN were not affected by different particle sizes of wheat bran (Table 4), but the resistin content tended to decrease (*p* = 0.05, *p* = 0.06, respectively). Four hours after feeding, the LEP and resistin contents of maternal serum were reduced (*p* < 0.05) in FWB-fed sows compared with CWB sows on day 90 of gestation.

**TABLE 4:** Effects of different particle sizes of wheat bran on maternal serum adipocytokines during gestation.

Item	CWB	FWB	*p*-value
**Fasting serum on day 90 of pregnancy**			
LEP, ng/ml	3.84 ± 0.49	3.65 ± 0.45	0.78
ADPN, μg/ml	3.90 ± 0.37	3.46 ± 0.50	0.50
Resistin, ng/ml	11.95 ± 1.01	8.82 ± 0.91	0.05
**Fasting serum on day 107 of pregnancy**			
LEP, ng/ml	4.19 ± 0.74	3.03 ± 0.37	0.21
ADPN, μg/ml	4.04 ± 0.62	3.52 ± 0.52	0.56
Resistin, ng/ml	10.66 ± 1.42	7.28 ± 0.38	0.06
**Serum after feeding on day 90 of pregnancy**			
LEP, ng/ml	4.50 ± 0.55	1.88 ± 0.21	<0.01
ADPN, μg/ml	3.26 ± 0.31	3.86 ± 0.44	0.30
Resistin, ng/ml	10.97 ± 0.93	8.10 ± 0.78	0.04
**Serum after feeding on day 107 of pregnancy**			
LEP, ng/ml	3.82 ± 0.73	2.53 ± 0.42	0.17
ADPN, μg/ml	4.10 ± 0.60	3.52 ± 0.61	0.52
Resistin, ng/ml	10.71 ± 1.37	6.93 ± 0.25	0.08

*CWB, a diet containing coarse wheat bran, with particle size of 605 μm; FWB, a diet containing fine wheat bran, with particle size of 438 μm; LEP, leptin; ADPN, adiponectin. Data are shown as mean ± SEM (n = 8).*

### Cytokines Content in Maternal Serum, Placenta, and Cord Blood

**FIGURE 3:**
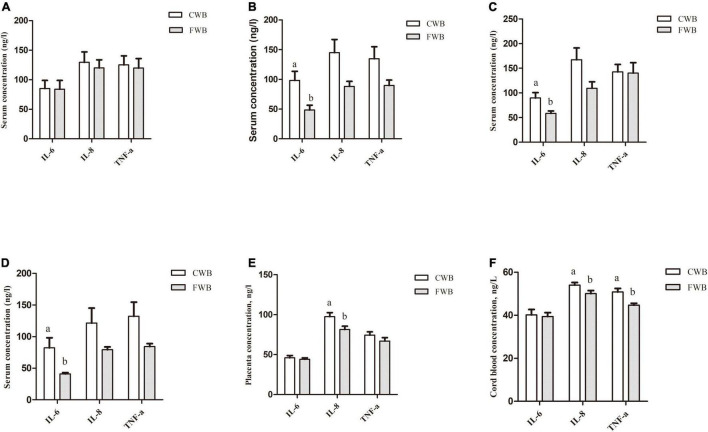
Effects of different particle sizes of wheat bran on levels of IL-6, IL-8, and TNF-α in maternal serum, placentae, and cord blood. **(A)** Fasting serum on day 90 of pregnancy. **(B)** Serum at 4 h after feeding on day 90 of pregnancy. **(C)** Fasting serum on day 107 of pregnancy. **(D)** Serum at 4 h after feeding on day 107 of pregnancy. **(E)** Placentae. **(F)** Cord blood. CWB, a diet containing coarse wheat bran, with particle size of 605 μm; FWB, a diet containing fine wheat bran, with particle size of 438 μm; IL-6, interleukin 6; IL-8, interleukin 8; TNF-α, tumor necrosis factor-α. Values are presented as mean ± SEM (*n* = 8). ^a,b^Means without common letters differ at *p* < 0.05.

During gestation day 90, fasting serum IL-6, IL-8, and TNF-α levels were not different between the CWB and FWB groups (*p* > 0.05) (Figure 3A). However, compared with the CWB group, the fasting serum level of IL-6 was reduced in FWB sows on day 107 of gestation (*p* < 0.05) (Figure 3C). Four hours after feeding, FWB sows had lower IL-6 (*p* < 0.05) in maternal serum on day 90 (Figure 3B) and day 107 (Figure 3D) of pregnancy. Likewise, compared with CWB sows, the concentrations of IL-8 were reduced (*p* < 0.05) in placentae and cord blood from FWB-fed sows (Figures 3E,F, respectively). Sows fed FWB had lower (*p* < 0.05) cord blood levels of TNF-α than CWB sows (Figure 3F).

### Fecal Microbiota Composition

**FIGURE 4:**
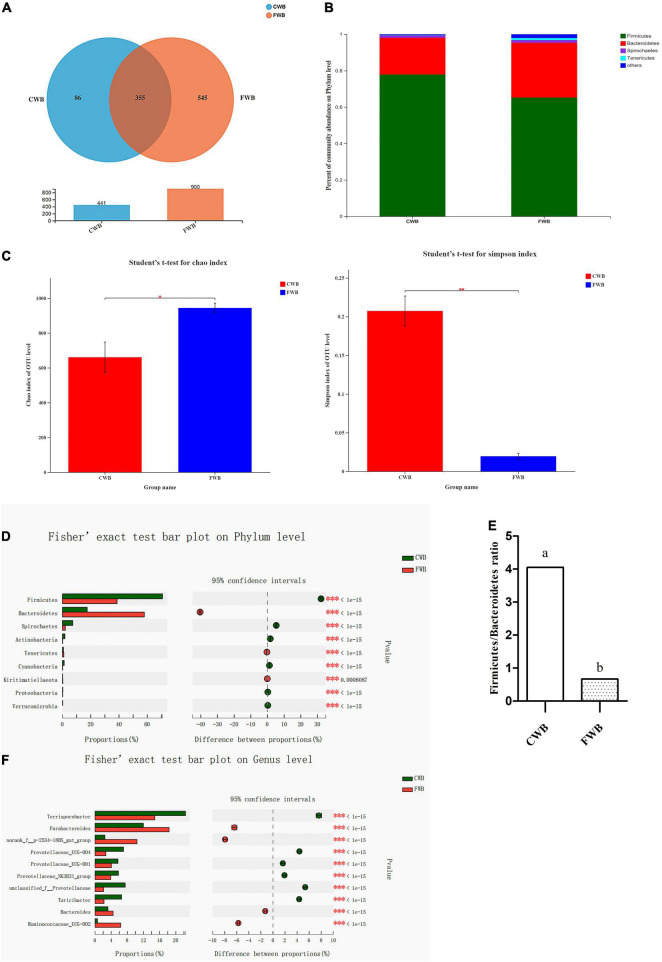
Effects of different particle sizes of wheat bran on maternal gut microbiota during gestation. **(A)** OTU Venn of two dietary treatments. **(B)** Percent of community abundance on phylum level; the results were analyzed by Student’s *t*-test and presented as mean values of different bacteria, *n* = 6. **(C)** The Chao index of bacterial community, *p* = 0.04; the Simpson index of bacterial community, *p* = 0.03. **(D)** The difference of microbiota on phylum level; the results analyzed by Fisher’s exact test, *n* = 6. **(E)** The ratio of *Firmicutes* to *Bacteroidetes*, the result presented as mean values. ^a,b^Means without common letters differ at *p* < 0.05. **(F)** The difference of microbiota on genus level, the results analyzed by Fisher’s exact test, *n* = 6; the *y*-axis represents the microbiota name at a certain classification level, and each column corresponding to the microbiota represents the relative abundance of the microbiota in each sample; the middle area is the set confidence interval, and the value corresponding to the circle point represents the relative abundance difference of the microbiota in the sample; the rightmost is *p*-value, *0.01 < *p* ≤ 0.05, ^**^0.001 < *p* ≤ 0.01, ^***^ *p* ≤ 0.001; CWB, a diet containing coarse wheat bran, with particle size of 605 μm; FWB, a diet containing fine wheat bran, with particle size of 438 μm.

Four hundred forty-one and 900 OTUs in the CWB and FWB groups were observed, respectively, of which 355 were shared between the two groups (Figure 4A). At the phylum level, *Firmicutes* and *Bacteroidetes* were the most abundant bacterial phyla, which accounted for about 90% of the total community, followed by *Spirochaetes* and *Tenericutes* (Figure 4B). Fecal microbial richness (Chao index) and diversity (Simpson index) were calculated (Figure 4C). The Chao index was significantly increased with fine wheat bran supplementation (*p* = 0.04). The Simpson index was lower for FWB-fed sows than CWB sows (*p* < 0.01). The relative abundances of *Firmicutes* and *Bacteroidetes* at the phylum level in FWB sows were very different from that in the CWB group. Sows fed FWB had a higher level of *Bacteroidetes* and a lower level of *Firmicutes* (*p* < 0.05) than CWB sows (Figure 4D). And the ratio of *Firmicutes* to *Bacteroidetes* (*F*/*B*) was significantly decreased in the FWB group (*p* < 0.05) (Figure 4E). At the genus level, the relative abundances of some specific genera, including *Parabacteroides*, *norank_f_p-2534-18B5_gut_group*, and *Ruminococcaceae_UCG-002*, were increased (*p* < 0.05) in sows from the FWB group; but compared with the CWB group, the FWB group had a lower relative abundance of *Terrisporobacter* (*p* < 0.05) (Figure 4F). The correlation heatmap showed the relationship between microbial genus and lipids, proinflammatory factors, and adipocytokines in serum. *Ruminococcaceae_UCG-002* and *Ruminococcaceae_UCG-005* were negatively correlated with TG level (Supplementary Figure 1A). The relative abundance of *Ruminococcaceae_UCG-002* and *Ruminococcaceae_UCG-005* was increased in the FWB group (*p* < 0.05) (Supplementary Figure 1B). *Clostridium_sensu_stricto_1* was positively related to IL-6 level, which decreased in FWB-fed sows (*p* < 0.05) (Supplementary Figures 2A,B). The *Ruminococcaceae_NK4A214_group* and *Christensenellaceae_R-7_group* showed highly negative correlation with LEP, ADPN, and resistin concentration in the serum of sows (Supplementary Figure 3A). The *Ruminococcaceae_NK4A214_group* and *Christensenellaceae_R-7_group* were increased (*p* < 0.05) in sows from the FWB group (Supplementary Figure 3B).

### Concentration of Short-Chain Fatty Acids From *in vitro* Fermentation and Feces

**FIGURE 5:**
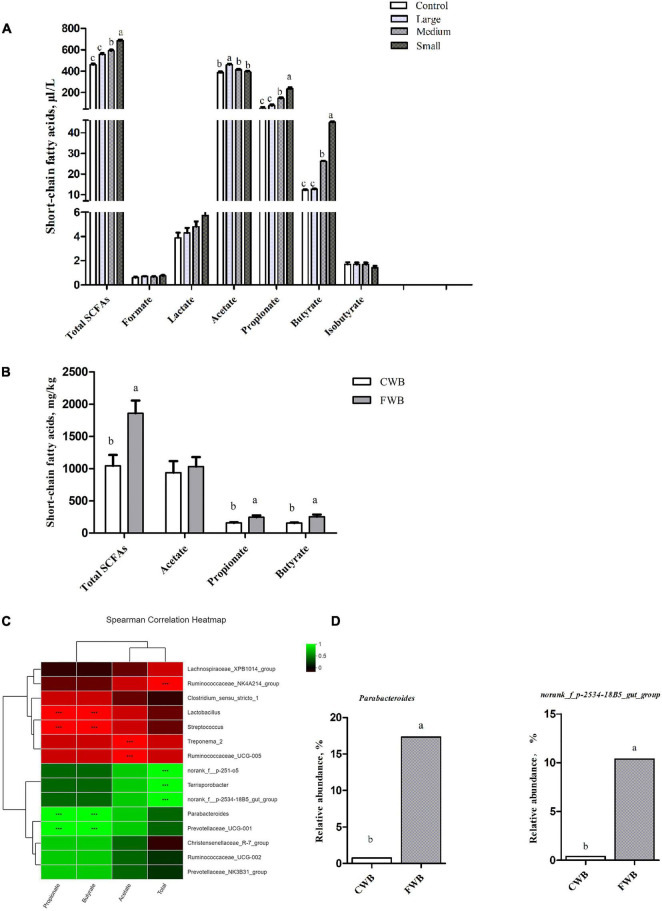
Content of SCFAs *in vitro* fermentation and in maternal feces, correlation heatmap of the 15 most abundant genera and SCFAs in maternal feces. **(A)** Content of SCFAs *in vitro* fermentation. **(B)** Concentrations of SCFAs in maternal feces. **(C)** X and Y axes present SCFAs and genus, respectively. The correlation coefficients (R) are shown in different colors on the right side of the legend. *0.01 < *p* ≤ 0.05, **0.001 < *P* ≤ 0.01, ***P ≤ 0.001. **(D)** The relative abundance of SCFA-related microbiota in two groups on the genus level. Control, no ground wheat bran; Large, wheat bran ground through a 2.0-mm sieve; Medium, wheat bran ground through a 1.5-mm sieve; Small, wheat bran ground through a 1.0-mm sieve; CWB, a diet containing coarse wheat bran, with particle size of 605 μm; FWB, a diet containing fine wheat bran, with particle size of 438 μm. Values are presented as mean ± SEM (*n* = 6). ^a,b^ Means without common letters differ at *p* < 0.05.

In *in vitro* fermentation, the total SCFAs content was significantly higher in the Small group than that in the Control, Large, and Medium groups (*p* < 0.05) (Figure 5A). Lactate, formate, and isobutyrate levels were similar among particle size treatments. Acetate was the most abundant SCFA, the concentration of which was similar among the Control, Medium, and Small groups, and was significantly higher in the Large group (*p* < 0.05). Propionate and butyrate contents were higher in the Small group than the Control, Medium, and Large groups (*p* < 0.05).

In feces, there was no difference in acetate content between the CWB and FWB groups, whereas total SCFAs, propionate, and butyrate were higher (*p* < 0.05) in FWB-fed sows than in CWB-fed sows (Figure 5B). The correlation heatmap showed the relationship between microbial genus and SCFAs. *norank_f_p-2534-18B5_gut_group* showed a highly positive correlation with total SCFAs, which was higher (*p* < 0.05) in FWB-fed sows than in CWB-fed sows; *Parabacterioides* was positively correlated with propionate and butyrate, which increased in FWB-fed sows (*p* < 0.05) (Figures 5C,D).

## Discussion

Dietary fiber could possess different physiological effects which are determined by its physicochemical properties. A large body of evidence demonstrated that the particle size of DF plays an important role in its physicochemical properties, which could affect its WHC, oil holding capacity, SC, and fermentation capacity (Stewart and Slavin, 2009; Li et al., 2020). In the present study, SC and WHC were improved with reducing particle size. The increase of specific surface area of wheat bran may lead to increased water absorption and improved SC. At the same time, the strong extrusion collision will lead to the exposure to numerous hydrophilic groups of cellulose and hemicellulose of DF, and these hydrophilic groups combine with water to increase WHC of wheat bran (Sarfaraz et al., 2017). Those changes of physicochemical properties of fine wheat bran will lead to different physiological effects, which may play a more effective role in maintaining appropriate body condition, lipid levels, and inflammatory profiles of sows during pregnancy.

Obesity during pregnancy is a key element of metabolic disorders which include a cluster of risk factors associated with increased risk of hyperlipidemia, hyperglycemia, and inflammation (Guelinckx et al., 2008). It is well known that DF is described as elements that affect energy homeostasis and body fat deposition (Islam et al., 2012). The intake of DF can play a role in the control of weight and fat mass gain through reducing energy intake, diminishing the efficiency of digestion and absorption of macromolecular nutrients in the gut (Sapkota et al., 2016), and influencing the production of SCFAs (Fang et al., 2019). Wheat bran is a source of IDF, which can help to protect gut health (McIntosh et al., 2003). But wheat bran rich in IDF is less effective on reducing lipid profiles and diet-induced obesity than oat bran rich in SDF (Han et al., 2015). In the current study, fine-grinding wheat bran had a higher level of SDF than the coarse one. Due to the strong pressure, shear force, friction force, and other comprehensive forces on wheat bran caused by grinding mechanical equipment in the process of grinding, the cellulose, hemicellulose, lignin, and other structures of the DF contained in wheat bran were destroyed leading to molecular chain breaks. Some insoluble components were degraded into soluble components, so the SDF content increased (Hemery et al., 2011). In the present study, FWB-fed sows had higher concentrations of SCFAs than that of CWB-fed sows. Meanwhile, fine wheat bran supplementation in gestational diets was beneficial in preventing excessive body weight gain and incremental backfat thickness throughout pregnancy. The reason for this result may be the fact that fine-grinding wheat bran had higher level of SDF, which was more likely to be fermented by hindgut microbiota to produce SCFAs, thus protecting against high-fat diet-induced obesity (Han et al., 2015).

Lipid metabolism is particularly affected during pregnancy. In late pregnancy, maternal hyperlipidemia is common to meet the demands of fetal growth and development (Herrera, 2000). However, excessive body weight gain and fat deposition in late pregnancy can cause dyslipidemia, mainly due to abnormally high TG content (Ghio et al., 2011). Excessive blood lipid levels in the host will increase the risk of insulin resistance and may be correlated with poor control of blood lipids in offspring (Li et al., 2015; Zheng et al., 2018). Meanwhile, elevated adiposity of pregnant sows could lead to increased maternal plasma TG concentrations but depressed TG in the cord blood due to downregulation of placental fatty acid transport-related proteins (Tian et al., 2018). The FWB group had less backfat thickness and a lower level of serum TG as well as placental TG compared with the CWB group, but the TG profiles in cord blood were inverted. These results might indicate that fine wheat bran could maintain proper body condition of sows during pregnancy and prevent abnormal lipid metabolism, which could be beneficial to lipid metabolism of sows. Proper body condition and lipid levels of sows could alleviate placental lipid abnormality, which could improve the reproductive performance of sows.

Adipocytokines including ADPN, resistin, and LEP have been found to be associated with obesity, insulin resistance, β-cell dysfunction, endothelial dysfunction, dyslipidemia, and inflammation (Filippatos et al., 2013). LEP is primarily produced by white adipose tissue, and circulating serum concentrations are directly associated with body fat mass and adipocyte size (HoTilg and Moschen, 2006). Elevated LEP levels in maternal blood are associated with maternal LEP resistance, metabolic disorders, and gestational diabetes during pregnancy (Bulló et al., 2003). Serum LEP level could exert obvious reduction due to less body weight gain and body fat accumulation with DF addition combined with a high-fat diet (Zhou et al., 2017). In line with these findings, maternal serum LEP level on day 90 of pregnancy was significantly reduced by fine wheat bran addition, which may have contributed to reduced body weight gain and fat accumulation. Resistin is a kind of adipokine that can resist insulin, increase blood sugar levels, and promote fat cell proliferation which facilitates obesity (Steppan et al., 2001). A higher level of resistin was observed in obese subjects than lean subjects (Sacks et al., 2004). In the current experiment, FWB-fed sows had a lower maternal serum resistin level, body weight, and fat accumulation on day 90 of pregnancy than CWB-fed sows. These observations indicated that fine wheat bran supplementation may play a role in controlling body weight gain, reducing fat deposition.

Pregnancy is a subclinical inflammatory process without obvious characteristics (Van Diepen et al., 2013). Inflammation is closely associated with lipid metabolism. Inflammation can aggravate the host’s lipid metabolism disorder (Feingold and Grunfeld, 1992). However, hyperlipoproteinemia can also promote the release of inflammatory factors. Lipolysis releases neutral and oxidized fatty acids that cause endothelial cell inflammation by stimulating formation of intracellular reactive oxygen species (Gouni et al., 1993; Challier et al., 2008; Wang et al., 2009). Dyslipidemia during pregnancy can lead to excessive inflammation in the placenta which is accompanied by the accumulation of multiple subsets of macrophages and production of proinflammatory mediators (Lazar, 2007). Inflammation exerts detrimental consequences for growth and development of the fetus. In the current study, we demonstrated that the FWB group had a lower level of IL-6 in maternal serum than that in the CWB group. The reduction in IL-6 is associated with the lower level of maternal serum TG and FFA in FWB-fed sows. Dietary supplementation with fine wheat bran to the gestation diet decreased levels of IL-8 in placentae and cord blood. Thus, the FWB diet may reduce inflammation of sow and placenta through the prevention of abnormal lipid metabolism. Simultaneously, piglets farrowed by FWB-fed sows were heavier at birth than piglets from CWB-fed sows. The results suggested that fine wheat bran supplementation could improve maternal reproductive performance by means of maintaining the normal environment of the placenta.

Sow fecal microorganisms were investigated. The interplay between intestinal microbiota and diet affects host physiology and metabolism (Tremaroli and Backhed, 2012). A large body of data has demonstrated that the gut microbiota structure is altered in individuals with obesity (Ley et al., 2006), which indicated that intestinal microbiota may be an important environmental factor leading to the development of metabolic diseases. Low microbial diversity is usually related to diseases such as inflammatory bowel disease (Nemoto et al., 2012) and obesity (Ley et al., 2006). The Chao index was significantly increased after fine wheat bran supplementation on day 90 of gestation. Simpson index, on the contrary, was smaller in the FWB group than that in the CWB group. This indicated that the FWB group had better microbial richness and diversity. The increased microbial richness and diversity could help maintain body weight and improve inflammatory responses during pregnancy, which is beneficial to sow health.

The abundance of specific phyla (*Firmicutes* and *Bacteroides*, 75% or more) in sow feces was consistent with previous studies on pigs (Han et al., 2018). Previous studies demonstrated that the number of *Bacteroides* was significantly reduced and *Firmicutes* was correspondingly increased in obese animals, thus changing the proportion of the primary gut microbiota (*Bacteroides* and *Firmicutes*) (Ley et al., 2006). On the phylum level, the relative abundance of *Bacteridetes* increased significantly, whereas the relative abundance of *Firmicutes* decreased significantly after supplementing with finely ground wheat bran. The relative abundance of *Bacteroidetes* in the intestine plays an important role in obesity and its related metabolic diseases such as type II diabetes (Johnson et al., 2016). Diet is the dominating driving factor of microbial composition (Turnbaugh et al., 2006). On the one hand, the increase of *Bacteridetes* may be due to the increase of SDF content in fine wheat bran. On the other hand, the microscopic structure of fine wheat bran may have changed and the contact area of microorganisms increased, which leads to the production of the enzyme by microorganisms to decompose fiber components more easily. The more fiber decomposition, the higher the SCFA content, which provides energy substrate for *Bacteridetes* growth. Fine wheat bran supplementation leads to suitable body condition of sows, which may be associated with changes in composition of gut microbiota. A decrease in the *F*/*B* was observed in sows fed with fine wheat bran diets. This finding is consistent with results of the research of Han et al. (2018) on whole-grain rice and wheat. Thus, the decrease of *F*/*B* should prevent obesity, which could prevent the occurrence of dyslipidemia and help maintain sow body condition.

*Parabacteroides*, a genus of gram-negative bacteria, is crucial for anti-inflammatory action and glucose and lipid metabolism (Kai et al., 2019). The relative abundance of *Parabacteroides* was negatively correlated with body mass index and inflammation (Kai et al., 2019). In the current study, the relative abundance of *Parabacteroides* was significantly increased in the FWB group, which could prevent excessive body weight gain, maintain normal blood lipid level, and alleviate inflammatory response of sows during pregnancy.

*Ruminococcaceae* has been well proven to be responsible for the breakdown of various polysaccharides and fibers (Shang et al., 2016). In addition, the relative abundance of *Ruminococcaceae* is significantly and negatively correlated with metabolic disease of liver (Owyang and Wu, 2014) and inflammation (Bajaj et al., 2012). The results in the current study indicated that *Ruminococcaceae_UCG-002* and *Ruminococcaceae_UCG-005* exhibited highly inverse correlation with TG. Besides, the relative abundance of *Ruminococcaceae_UCG-002* and *Ruminococcaceae_UCG-005* was increased in the FWB group. Hence, the increase of *Ruminococcaceae_UCG-002* and *Ruminococcaceae_UCG-005* may be able to maintain normal liver function and normal lipid metabolism, so as to maintain appropriate blood lipid levels during pregnancy of sows. *Clostridium_sensu_stricto_1* is generally regarded as pathogenic bacteria, which is highly related to inflammation (Yang et al., 2019). And the decreased relative abundance of *Clostridium_sensu_stricto_1* can reduce proinflammatory factor levels in sows (Shang et al., 2021). In the present study, there was a significant positive correlation between *Clostridium_sensu_stricto_1* and proinflammatory factors. Meanwhile, the relative abundance of *Clostridium_sensu_stricto_1* was decreased in the FWB group. As a result, the decrease in relative abundance of *Clostridium_sensu_stricto_1* may be responsible for the decreased levels of proinflammatory factors in sows’ blood. Therefore, it can relieve the inflammatory state of sows during pregnancy. *Christensenellaceae* plays an important role in the host health (Waters and Ley, 2019). And *Christensenellaceae* is highly negatively correlated with obesity (Alemán et al., 2018), visceral fat mass (Beaumont et al., 2016), and TG levels (Fu et al., 2015). In the present research, *Christensenellaceae_R-7_group* was negatively correlated with LEP and resistin. The relative abundance of *Christensenellaceae_R-7_group* was higher in the FWB group than that in the CWB group. The circulating serum concentrations of LEP are directly associated with body fat mass and adipocyte size (HoTilg and Moschen, 2006). And resistin can promote fat cell proliferation which facilitates obesity (Steppan et al., 2001). Therefore, the increased relative abundance of *Christensenellaceae_R-7_group* is beneficial to maintaining suitable body fat deposition and body weight of sows.

Microbial community structural and metabolic responses are affected by the DF structure (Thakkar et al., 2020). The study detected fermentability of wheat bran with different particle sizes *in vitro*. The results found that fine wheat bran produced more total SCFAs, propionate, and butyrate by microbial fermentation, which is consistent with the report of Stewart and Slavin (2009). And Thakkar et al. (2020) demonstrated that the smallest size fraction of maize bran was much more extensively fermented, eliciting the highest amount of all three SCFAs (acetate, propionate, and butyrate). This difference, on the one hand, may be due to the increase in accessible surface area as particle size decreases, which provides bacterial enzymes with a larger contact area to ferment carbohydrates. On the other hand, SDF is easier to be fermented than IDF and consequently has a larger effect on bacterial metabolism (Gråsten et al., 2002). The results demonstrated that an addition in SDF level and a reduction in IDF level in fine wheat bran is correlated with increased SCFAs content. Variation of fermentation characteristics of finely ground wheat bran may contribute to changes of microbes and their fermentation products in the hindgut of sows.

Short-chain fatty acids are the main metabolites of microbial fermentation of DF in the hindgut, which modulates host physiological responses, including lipid metabolism and the immune system (Fukuda et al., 2011). Butyrate is almost completely used by colonocytes as their preferred energy substrate (Pryde et al., 2002). Propionate is associated with positive effects on metabolic health, such as lowered serum cholesterol (Tomomi et al., 2018). The present study demonstrated that the FWB group had a higher concentration of total SCFAs, propionate, and butyrate than the CWB group. Besides, the changes of SCFAs concentration were consistent with that of *in vitro* fermentation. Consequently, increased total SCFAs, propionate, and butyrate are beneficial to maintain blood lipid and alleviate the inflammatory state of sows. In addition, the results found that *Parabacteroides* was significantly positively correlated with propionate and butyrate. And in the FWB group, the abundance of *Parabacteroides* was significantly increased. Thus, the interaction between microbes and SCFAs is crucial.

## Conclusion

Finely ground wheat bran had a better SC, WHC, and fermentability. Dietary supplementation with fine wheat bran changed the gut microbiota structure and enhanced the concentration of SCFAs, which improved pregnancy weight gain, lipid metabolism, and maternal anti-inflammation in sows.

## Data Availability Statement

The datasets presented in this study can be found in online repositories. The names of the repository/repositories and accession number(s) can be found below: https://submit.ncbi.nlm.nih.gov/subs/bioproject/. ID PRJ NA 692550.

## Ethics Statement

The animal study was reviewed and approved by Animal Care and Use Committee of China Agricultural University. Written informed consent was obtained from the owners for the participation of their animals in this study.

## Author Contributions

FW contributed to designing the studies and providing financial support. ZW contributed to doing animal experiment, executed the lab analysis, performed the statistical analysis, and prepared the manuscript. YC, WW, and CH assisted in preparing the manuscript draft. All the authors have read and approved the final manuscript. YH and LJ revised the manuscripts.

## Conflict of Interest

The authors declare that the research was conducted in the absence of any commercial or financial relationships that could be construed as a potential conflict of interest.

## Publisher’s Note

All claims expressed in this article are solely those of the authors and do not necessarily represent those of their affiliated organizations, or those of the publisher, the editors and the reviewers. Any product that may be evaluated in this article, or claim that may be made by its manufacturer, is not guaranteed or endorsed by the publisher.

## Abbreviations

ADPN: adiponectin
CM: chylomicrons
CP: crude protein
CWB: a diet containing coarse wheat bran
DF: dietary fiber
DM: dry matter
*F/B*: the ratio of *Firmicutes* to *Bacteroidetes*
FFA: free fatty acid
FWB: a diet containing fine wheat bran
IDF: insoluble dietary fiber
IL-6: interleukin 6
IL-8: interleukin 8
LEP: leptin
NDF: neutral detergent fiber
SC: swelling capacity
SCFAs: short-chain fatty acids
SDF: soluble dietary fiber
TDF: total dietary fiber
TC: total cholesterol
TG: triglyceride
TNF-α: tumor necrosis factor-α
VLDL: very low-density lipoprotein
WHC: water-holding capacity.
