# Supplementary material for: Dietary Supplementation With Fine-Grinding Wheat Bran Improves Lipid Metabolism and Inflammatory Response via Modulating the Gut Microbiota Structure in Pregnant Sow
Source: Front Microbiol. 2022 Mar 24;13:835950. doi: 10.3389/fmicb.2022.835950 (PMC8999112; doi:10.3389/fmicb.2022.835950)
Supplement: Supplementary file 1 [file Data_Sheet_1.docx]

**Supplementary data**

**Supplemental Table 1.** Ingredient composition and nutrient levels of experimental diets (%, as-fed basis).

| Item | Mating - day 90 of gestation | | Day 91 of gestation to parturition | |
| --- | --- | --- | --- | --- |
|  | CWB^3^ | FWB^4^ | CWB | FWB |
| Corn | 68.27 | 68.27 | 65.82 | 65.82 |
| Soybean meal | 9.50 | 9.50 | 10.00 | 10.00 |
| Coarse wheat bran^1^ | 20.00 |  | 20.00 |  |
| Fine wheat bran^2^ |  | 20.00 |  | 20.00 |
| Soybean oil |  |  | 2.00 | 2.00 |
| Dicalcium Phosphate | 0.38 | 0.38 | 0.38 | 0.38 |
| Limestone | 1.05 | 1.05 | 1.00 | 1.00 |
| Salt | 0.30 | 0.30 | 0.30 | 0.30 |
| Vitamin and mineral premix^5^ | 0.50 | 0.50 | 0.50 | 0.50 |
| Calculated composition |  |  |  |  |
| ME, Kcal/kg | 3190 | 3190 | 3292 | 3292 |
| SID lysine | 0.61 | 0.61 | 0.69 | 0.69 |
| Calcium | 0.55 | 0.55 | 0.78 | 0.78 |
| Total phosphorus | 0.54 | 0.54 | 0.58 | 0.58 |
| Analyzed composition |  |  |  |  |
| Crude protein | 13.11 | 13.19 | 13.74 | 13.81 |
| Neutral detergent fiber | 24.88 | 24.12 | 24.88 | 24.12 |
| Total dietary fiber | 20.53 | 20.50 | 20.53 | 20.50 |
| Soluble dietary fiber | 2.20 | 2.79 | 2.20 | 2.79 |
| Insoluble dietary fiber | 18.28 | 17.55 | 18.28 | 17.55 |

^1^ coarse wheat bran, with particle size of 605 μm. Analyzed coarse wheat bran (as fed-basis): DM, 87.37%; Ash, 5.02%; CP, 16.93%; NDF, 51.65%; ADF, 13.23%; TDF, 44.56%; SDF, 4.01%; IDF, 39.76%

^2^ fine wheat bran, with particle size of 438 μm. Analyzed fine wheat bran (as fed-basis): DM, 86.98%; Ash, 4.55%; CP, 16.79%; NDF, 49.11%; ADF, 13.01%; TDF, 43.84%; SDF, 5.98%; IDF, 37.94%

^3^ CWB: a diet contained coarse wheat bran with particle size of 605 μm

^4^ FWB: a diet contained fine wheat bran with particle size of 438 μm

^5^ Premix provided per kilogram of diet: vitamin A, 11,000 IU; vitamin D3, 1,500 IU; vitamin E, 15 IU; vitamin K3, 1.6 mg; vitamin B1, 1.5 mg; vitamin B2, 3.0 mg; vitamin B6, 1.5 mg; vitamin B12, 0.015 mg; niacin, 22.5 mg; D-pantothenic acid, 15 mg; biotic, 0.2 mg; Fe, 85 mg; Cu, 75 mg; Mn, 35 mg; I, 0.5 mg

**Supplemental Fig 1.** The correlation heatmap of the 15 most abundant genera and lipids. A: X and Y axes present lipids and genus, respectively. The correlation coeffificients (R) are shown in different colors on the right side of the legend. The value of 0.01 < *p* ≤ 0.05 is marked with * and *p* < 0.01 is marked with **; B: The relative abundance at genus level negatively correlated with lipids. ^a,b^ means without common letters differ at *p* < 0.05.

**Supplemental Fig 2.** The correlation heatmap of the 15 most abundant genera and inflammation cytokines. A: X and Y axes present inflammation cytokines and genus, respectively. The correlation coeffificients (R) are shown in different colors on the right side of the legend. The value of 0.01 < p ≤ 0.05 is marked with *, 0.001 < p ≤ 0.01 is marked with ** and p ≤ 0.001 is marked with ***; B: The relative abundance at genus level positively correlated with inflammatory factors. ^a,b^ means without common letters differ at *p* < 0.05.

**Supplemental Fig 3.** The correlation heatmap of the 15 most abundant genera and adipocytokines. A: X and Y axes present adipocytokines and genus, respectively. The correlation coeffificients (R) are shown in different colors on the right side of the legend. The value of 0.01 < *p* ≤ 0.05 is marked with * and *p* < 0.01 is marked with **; B: The relative abundance at genus level negatively correlated with adipocytokines. ^a,b^ means without common letters differ at *p* < 0.05.
