# Supplementary figures and images for: Dietary Supplementation With Fine-Grinding Wheat Bran Improves Lipid Metabolism and Inflammatory Response via Modulating the Gut Microbiota Structure in Pregnant Sow
Source: Front Microbiol. 2022 Mar 24;13:835950. doi: 10.3389/fmicb.2022.835950 (PMC8999112; doi:10.3389/fmicb.2022.835950)

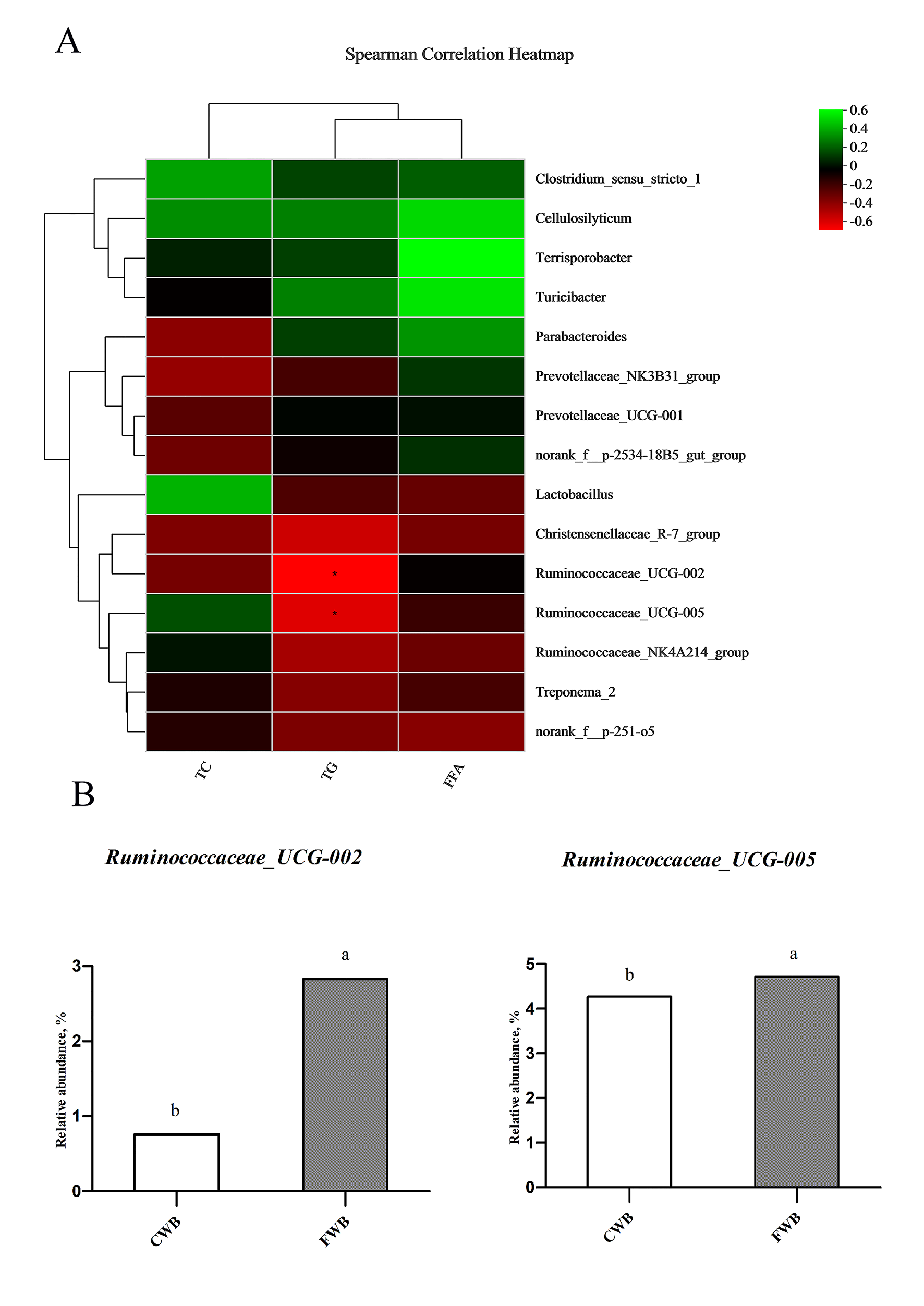

Supplement: Supplementary file 2 [file Image_1.TIF]

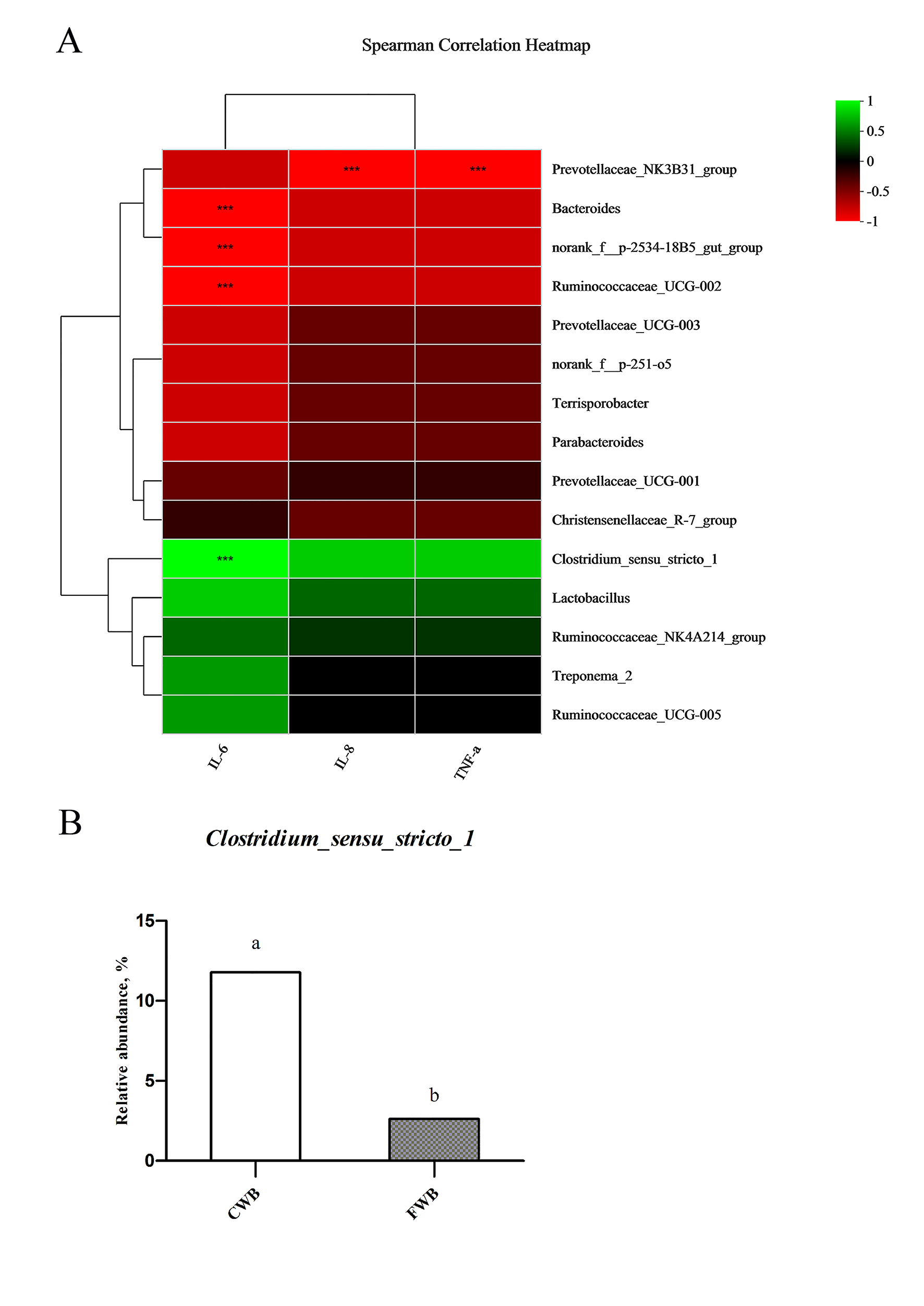

Supplement: Supplementary file 3 [file Image_2.TIF]

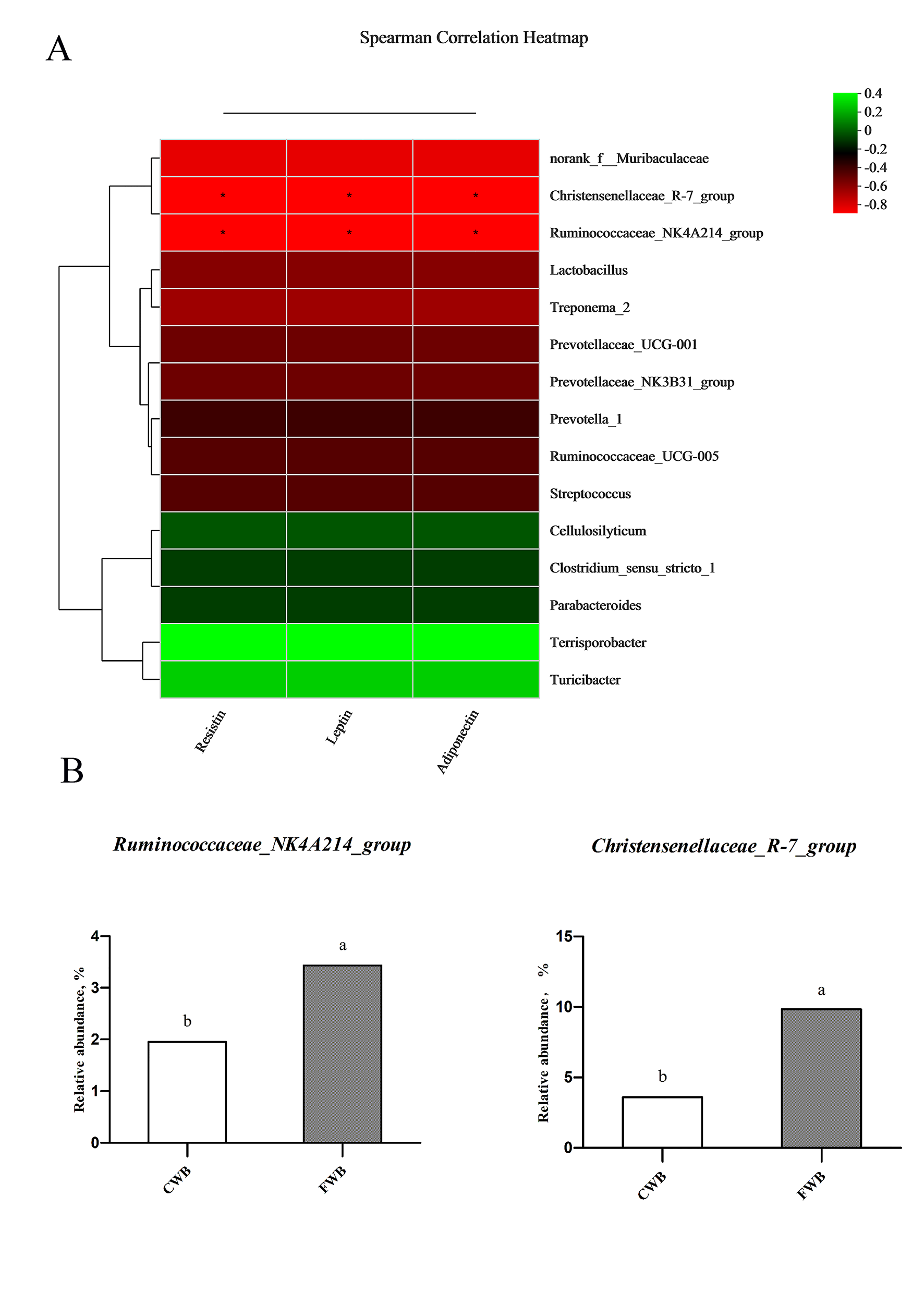

Supplement: Supplementary file 4 [file Image_3.TIF]
